# Prolonged Grief Disorder and the Cultural Crisis

**DOI:** 10.3389/fpsyg.2019.02982

**Published:** 2020-01-10

**Authors:** Eva-Maria Stelzer, Ningning Zhou, Andreas Maercker, Mary-Frances O’Connor, Clare Killikelly

**Affiliations:** ^1^Division of Psychopathology and Clinical Intervention, University of Zurich, Zurich, Switzerland; ^2^Department of Psychology, University of Arizona, Tucson, AZ, United States; ^3^Faculty of Psychology, Beijing Normal University, Beijing, China

**Keywords:** prolonged grief disorder, Asian, cross-culture, prevalence, bereavement

## Abstract

Prolonged grief disorder (PGD) is included as a new mental health disorder in the 11th edition of the International Classification of Diseases (ICD-11). Understandably, this has boosted research efforts to investigate this newcomer to psychopathology. However, the use of different diagnostic algorithms has resulted in substantially different prevalence rates both within and across cultural groups. Furthermore, global applicability of the new criteria outside of the Global North has not been yet been established. This perspective presents key findings from Asian research groups and discusses the roadblocks to unified PGD research, including the heterogeneric use of diagnostic algorithms and the lack of cultural compatibility of ICD-11 items. The authors discuss the key issues and address implications for practice.

## Highlights

- The new ICD-11 Prolonged Grief Disorder (PGD) criteria are conceptualized in terms of the World Health Organization’s prioritization of international applicability.
- However, significant barriers exist to extrapolate prevalence rates within and across culture, and to establish global applicability.
- Barriers to unified PGD research include, among others, the use of different diagnostic algorithms and a lack of research regarding the cultural specificity of current ICD-11 items leading to an evolving cultural crisis of potential misestimation of prevalence rates.
- Emerging research from Asia confirms the worldwide heterogeneity in the use of PGD algorithms and the extent of the crisis.

## Introduction

The field of grief and bereavement is at a turning point. For the first time, prolonged grief disorder (PGD) is included as a new mental health disorder in the World Health Organization’s (WHO) 11th edition of the International Classification of Diseases (ICD-11) (World Health Organization [WHO], 2018). This presents the opportunity for igniting research into assessment and treatment and providing clinicians with strong and unified groundwork for the validity of this disorder. However, currently there are some significant roadblocks to concerted PGD research. Firstly, the new formulation of PGD is preceded by several different disorder definitions that are currently in use. This has led to a wide variation in the use of diagnostic algorithms yielding substantially different prevalence rates, also across different cultural groups. This is unaided by the fact that the ICD-11 has not formally included a recommended diagnostic algorithm but rather applies a typological approach for diagnoses. Secondly, the clinical description of PGD for the ICD-11 is newly conceptualized following the WHO’s prioritization of global applicability. PGD is characterized by core symptoms of longing for or preoccupation with deceased, significant symptoms of emotional distress, in addition to key cultural caveats, i.e., the duration of disorder must violate expected social and cultural norms (Killikelly and Maercker, 2017; World Health Organization [WHO], 2018). However, the global applicability of diagnostic criteria outside of the Global North has not yet been established. For example, the content of the specific items is largely derived from the Global North (Prigerson et al., 2009; Maciejewski et al., 2016). Several research groups across Asia are spearheading a new wave of research that is exploring and evaluating PGD. This perspective presents the latest research from Asia that uses the PGD criteria (as opposed to complicated grief or persistent complex bereavement disorder) and the new ICD-11 formulation of PGD. Here we present key findings from East-Asian research groups that expose the widespread difficulty with the heterogenic use of diagnostic algorithms and also challenge surface-level cultural compatibility of the specific ICD-11 items. We propose that the continued use of different diagnostic algorithms will undermine the field and lead to a crisis in terms of inaccurate prevalence rates of PGD, particularly in cross cultural research.

## Prevalence Crisis

Around the world, prevalence rates for PGD have been examined using several different sets of diagnostic algorithms. Most predominantly the PGDPlos criteria (Prigerson et al., 2009), ICD-11 PGD criteria (as closely as possible modeled after ICD-11; Maciejewski et al., 2016; Killikelly and Maercker, 2017), criteria from the Inventory of Complicated Grief (ICG) (Prigerson et al., 1995; Prigerson and Maciejewski, 2005; Shear et al., 2005), and variations of these criteria are used (Table 1). Not surprisingly, the use of different criteria sets has resulted in different prevalence rates of diagnosis. Using the PGDPlos criteria (Prigerson et al., 2009), prevalence rates for PGD in East Asian studies range from 1.8% for the general population (He et al., 2014) to 21.73% for those who lost their only child (Shi et al., 2019). In contrast, a recent study that applied the new ICD-11 PGD criteria following Killikelly and Maercker (2017) and Maciejewski et al. (2016) documents prevalence rates of 38.7 and 31.5% for Chinese Shidu parents (Zhou et al., under review). Finally, a range of other variations on these criteria have revealed different prevalence rates. For instance, studies operationalizing PGD in terms of the CG formulation (Prigerson et al., 1995; Prigerson and Maciejewski, 2005; Shear et al., 2005) find prevalence rates ranging from 9.8 (Tsutsui et al., 2014) to 35% (Yu et al., 2016). And according to Yu et al. (2017) almost half of their sample (47.2) met criteria for PGD applying yet another diagnostic algorithm to dichotomize individuals in PGD vs. non-PGD clusters.

**TABLE 1:** Common diagnostic algorithms for the assessment of PGD and their prevalence rates in East-Asian samples.

**Diagnostic criteria**	**Algorithm**	**Study findings**			
		**Authors**	**Prevalence rates (%)**	**Sample**	**Sample size (*N*)^1^**
PGDPlos (Prigerson et al., 2009)	Experience of yearning Cognitive, emotional, and behavioral symptoms: ≥5 Time since death: ≥6 months Significant impairment	He et al., 2014	1.8	Chinese sample; general population	445
		Tsai et al., 2018	5.2 (6 months post loss); 2.3 (13 months post loss)	Taiwanese caregivers	388 (6 months) 354 (13 months)
		Yi et al., 2018	8.47	Chinese earthquake survivors	1464
		Li and Prigerson, 2016	13.9	Chinese sample	1099
		Zhou et al., 2018	16.20	Chinese Shidu parents	536
		Comtesse and Rosner, 2019	20.2	Middle-Eastern asylum seekers	99
		Zhou et al., under review	20.9	Chinese Shidu parents	961
		Shi et al., 2019	21.73	Chinese Shidu parents	308
ICD-11 PGD (Killikelly and Maercker, 2017)	Experience of separation distress: yearning or preoccupation Experience of intense emotional pain symptoms: ≥1 Time since death: >6 months Significant impairment	Zhou et al., under review	38.7	Chinese Shidu parents	961
ICD-11 PGD criteria with additional accessory symptoms (Maciejewski et al., 2016)	Experience of yearning or preoccupation Syndrome severity: ≥3 items Time since death:>6 months Significant impairment	Zhou et al., under review	31.5	Chinese Shidu parents	961
CG formulation (e.g., (Prigerson et al., 1995; Prigerson and Maciejewski, 2005; Shear et al., 2005)	Cut-off score of >25 (Prigerson et al., 1995) on Inventory of Complicated Grief defined as PGD-positive	Tsutsui et al., 2014	9.8	Japanese hospital workers after earthquake	82
	Prigerson and Maciejewski, 2005 Experience of yearning Cognitive, emotional, and behavioral symptoms: ≥4 Time since death: ≥6 months Significant impairment	Rajkumar et al., 2015	14.2 for tsunami survivors 25.9 for bereaved survivors	South Indian tsunami survivors	643 tsunami survivors; 351 bereaved survivors
	Cut-off score of ≥30 (Shear et al., 2005) on Inventory of Complicated Grief defined as PGD	Yu et al., 2016	35	Chinese widow(er)s	120
Other criteria	High PGD group: Rating of 3 or more on ≥3 PG-13 items with symptoms experienced at least once a week. Low PGD group: Rating of 2 or less on <3 PG-13 items with symptoms experienced less than once a week	Yu et al., 2017	47.2 in high PGD group	Chinese sample	72

*^1^If information was available, the number of participants used for prevalence estimation is presented (rather than entire sample size of a particular study).*

A similar variation can be found for research conducted in the Global North (Bonanno and Malgaroli, 2019; O’Connor et al., 2019). For instance, in a sample of US widow(er)s, Bonanno and Malgaroli (2019) reported prevalence rates of 4.8–12.2% when comparing different diagnostic algorithms for ICD-11 PGD criteria with varying numbers of accessory symptom items. The use of these different diagnostic algorithms makes it very difficult to extrapolate prevalence rates of PGD across Asian countries and to compare these rates with prevalence rates in the Global North, posing a significant risk of over- or underestimating the rate of disorder. For instance, a higher diagnostic threshold for assessing pathological grief has been suggested for Chinese samples (Li and Prigerson, 2016). However, this may only be the case when using the CG formulation applied by Li and Prigerson (2016). Furthermore, comparability and conclusions are limited as algorithms are derived from a number of scales, assessment procedures (self-report vs. clinical interviews), and item responses (score of at least three vs. four) to assess symptom endorsement.

## Beyond Prevalence

In addition, it is unclear if the prevalence rates indicate the most valid symptom expression. Although in both Chinese individuals and samples from the Global North, yearning or longing for the deceased, indicative of separation-related distress, constitute the hallmark symptom for disordered grief (Prigerson et al., 2009; He et al., 2014; Li and Prigerson, 2016; Xiu et al., 2016), there may be other culturally bound symptoms of grief that are currently not included in the ICD-11 PGD criteria (Killikelly et al., 2018). There are several examples of how mental health measurement derived from within the cultural group may inform the validity of the assessment. In a foundational series of studies, Hinton et al. (2013) explored symptoms of PGD in Cambodian refugees using a gold standard grief scale from the Global North [Prolonged Grief-13 (PG-13), Prigerson et al., 2009]. Interestingly they also developed a culturally sensitive measure of grief (CSM-G) and compared the rates of distress using these two scales. The CSM-G measure included reference to the somatic and bodily experiences of distress that are not included in the PG-13. According to the PG-13, 8% of participants experienced significant grief distress, whereas the locally derived scale revealed that over 31% of participants met criteria for severe distress. These contrasting results between etic (measures developed outside of the culture) and emic (measures developed from within the culture) scales have been consistently found across different cultural groups and for different mental health disorders (Patel et al., 1997; Fernando, 2012; Rasmussen et al., 2014; Kim et al., 2017). If the ICD-11 guidelines for PGD seek global applicability, guidance on specific adaptations for different cultural groups should be provided to improve the cross-cultural validity (Bäärnhielm et al., 2015; Lewis-Fernández et al., 2017; Smid et al., 2018).

Additionally, features of the ICD-11 PGD criteria may be more or less common for certain cultural subgroups and thus should be weighted accordingly. We explored the cross-cultural utility and applicability of the ICD-11 PGD criteria among Chinese and German-speaking health-care providers (Stelzer et al., in press). While health-care providers were generally aligned with the diagnostic criteria, they also identified symptoms currently missing in the ICD-11 such as the experience of somatic/physical symptoms after loss, and symptoms that should be removed from the diagnostic criteria for certain cultural groups (e.g., anger, sadness, or loss of self in China). Cultural variations also became apparent for the time-criterion. Here, Chinese health-care providers suggested to extend the criterion to match the culturally prescribed mourning period. Such differences between the Global North and Asian samples with regard to endorsement of specific accessory symptoms have also been noted in quantitative studies (Li and Prigerson, 2016; Xiu et al., 2016). In a cross-cultural comparison of bereaved parents, the Chinese sample reported a stronger sense that life is empty or meaningless whereas Swiss parents suffered from more severe grief-related preoccupation (Xiu et al., 2016). Similarly, trauma-related distress and grief hallucinations are particularly common in Chinese samples (Li and Prigerson, 2016) but less strongly endorsed in the Global North. And of course, sociocultural and logistical barriers such as a lack of culture-sensitive assessment tools and unfamiliarity with diagnostic systems further challenge the global applicability of ICD-11 PGD criteria (Stelzer et al., in press). Overall, these findings highlight the need to consider cultural factors and specific symptoms when assessing and generalizing PGD criteria across cultural groups.

## Discussion

Many cultural factors, working invisibly in the background, affect the way symptoms are expressed, perceived, assessed, interpreted, and documented. Religious beliefs and practices, for instance, are strongly related to culture and may account for some of the variations in grieving processes (Lobar et al., 2006; Beyers, 2017). Influenced by beliefs such as fatalism [e.g., “Life and death are decreed by fate” (Yang, 1980)], Chinese bereaved seldomly share their grief with others and when Chinese people comfort the bereaved they often do so by saying “Restrain your grief and accord with inevitable changes”. The subtle influences of cultural context are observable when someone outside the culture disrupts the invisible veil that hides them. This brings to light important cultural differences that have not been discussed in the scientific literature. As empirical research forms the basis for clinical decision-making, validation of grief reactions across cultures is essential prior to generalizing symptom clusters – especially when trying to provide adequate support and comfort to the bereaved. The initial period when guidelines are first applied by clinicians is critical – before years of practice have solidified them, making them more resistant to change. Since the *expression of grief varies by culture, despite the universal experience of grief*, it is vitally important that we detect these variations early as we apply diagnostic criteria. In recent years, an increasing number of empirical studies (of Chinese origin in particular) have explored grief responses in samples beyond the Global North and added to a growing body of knowledge regarding prevalence rates of pathological grief. But despite these efforts from researchers and WHO stakeholders, we are far from establishing global applicability of PGD. The heterogeneity of diagnostic algorithms used by researchers and clinicians resulting in different prevalence rates within and across cultural groups constitutes one of the major challenges of cultural compatibility. If researchers keep using different diagnostic approaches to assess cases of PGD, conclusions and comparisons regarding prevalence rates and cross-cultural applicability are hampered.

We therefore recommend that expert researchers and clinicians in the field come together to decide on a consensus PGD algorithm that can be used across research groups and countries. The new wave of research emerging from Asian countries should stimulate collaboration to explore cross-cultural similarities and differences in the presentation of grief. With the novum of having disordered grief included in classification systems for mental health disorders, it is critical to no longer presume a universality of grief responses but instead move toward a culturally integrated grief framework. Further research can examine this by collecting open-ended written responses to questions or interviews about grief reactions in a culture and generating culture-specific grief items (Shoeb et al., 2007; Yin et al., 2017). Additionally, cultural clinical interviews could be employed to grasp a full understanding about grief severity. For example, recently, researchers in the Netherlands have developed the Grief and Bereavement cultural interview that aims to provide an in-depth assessment of the cultural context underlying prolonged grief distress (Smid et al., 2018). This 10-question interview could supplement a standard grief scale such as the PG-13 or the new International PGD scale (Killikelly et al., in preparation) to better guide the severity of the diagnosis and treatment planning.

Although there is no gold standard procedure for cross-cultural measurement of PGD and it is currently unknown how feasible such an assessment might be for grief, several researchers have confirmed that culturally informed mental health assessment is feasible, useful, and improves the validity of mental health diagnosis (Aggarwal et al., 2014; Kirmayer et al., 2014; Lewis-Fernández et al., 2017). Methodologically sound investigations and systematic cross-cultural comparisons can help identify in what ways grief phenomena are universal and culture specific. This knowledge, in turn, can influence existing bereavement models and clinical practice allowing researchers and clinicians to take individuals’ cultural identities into account when providing assistance and comfort to culturally diverse populations. It is important for researchers and clinicians to share knowledge and develop a consensus on required cultural caveats and culturally specific symptoms to improve assessment and treatment of disordered grief.

## Author Contributions

E-MS constructed the structure of the perspective and wrote most of it. NZ searched the articles conducted in China and reviewed the results of the Chinese studies. CK proposed the idea and directed the whole process of the writing. AM and M-FO’C read and revised the manuscript in detail.

## Conflict of Interest

The authors declare that the research was conducted in the absence of any commercial or financial relationships that could be construed as a potential conflict of interest.
